# Supplementary material for: Papain Suppresses Atopic Skin Inflammation through Anti-Inflammatory Activities Using In Vitro and In Vivo Models
Source: Antioxidants (Basel). 2024 Jul 30;13(8):928. doi: 10.3390/antiox13080928 (PMC11351312; doi:10.3390/antiox13080928)
Supplement: Supplementary file 1 [file antioxidants-13-00928-s001.zip › antioxidants-3109935-supplementary.pdf]

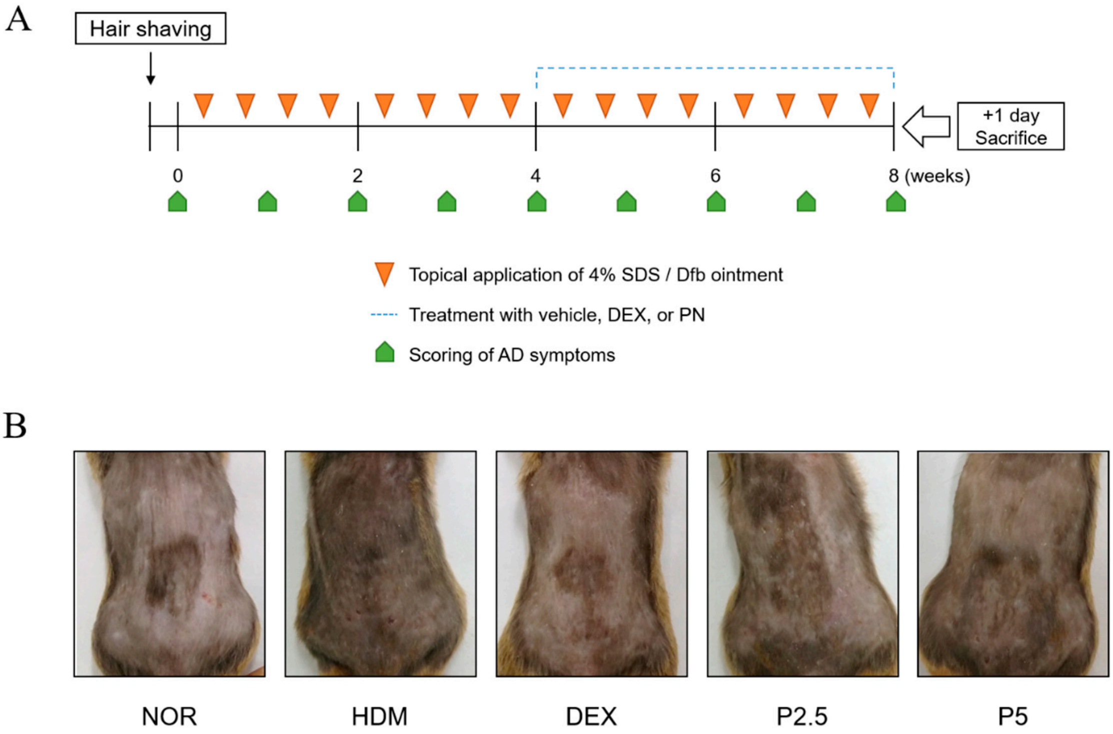

Figure S1. Effects of PN on the clinical features of Dfb-induced AD skin in NC/Nga mice. (A) The scheme of experiment. (B) Clinical features of AD-skin symptoms.
